# Chemotherapeutic Loading and Delivery of Patient-Derived Extracellular Vesicles Are Influenced by Colorectal Cancer Disease Stage and Protein Corona

**DOI:** 10.3390/pharmaceutics18060740

**Published:** 2026-06-15

**Authors:** Otman Saud, Dallal Blidi, Emily Hayes, Celine Souilhol, Rawan Maani, Alice Johnson, Keith Chapple, Nick Peake

**Affiliations:** 1 Biomolecular Sciences Research Centre, Sheffield Hallam University, Sheffield S1 1WB, UK; 2 Colorectal Surgical Unit, Sheffield Teaching Hospitals NHS Foundation Trust, Sheffield S10 2JF, UK

**Keywords:** colorectal cancer, extracellular vesicle, doxorubicin, autologous, plasma

## Abstract

**Background/Objectives**: Colorectal cancer (CRC) remains a leading cause of cancer-related mortality, with poor outcomes in advanced stages and significant limitations in current chemotherapy regimens due to systemic toxicity. Extracellular vesicles (EVs) have emerged as promising natural drug delivery vehicles, offering the potential for targeted, less toxic therapies. This study investigates the feasibility of using autologous, patient-derived EVs as a delivery system for the chemotherapeutic agent doxorubicin, focusing on how disease stage and the EV protein corona influence loading and delivery efficiency. **Methods**: EVs were isolated from plasma and tissue samples of CRC patients at different disease stages, as well as from healthy controls, demonstrating successful isolation and characterisation of EVs, with distinct profiles across different sources. **Results**: Doxorubicin loading into EVs was significantly higher in CRC patient-derived EVs compared to healthy controls, and tissue-derived EVs yielded higher quantities of drug-loaded particles. Delivery of doxorubicin-loaded EVs to recipient CRC cell lines (SW480 and SW620) revealed that disease stage impacts both EV uptake and drug delivery, with late-stage EVs showing reduced uptake and delivery efficiency. The protein corona, known to coat circulating EVs, was found to influence drug loading and delivery. Pre-treatment of cell line-derived EVs with plasma proteins enhanced EV uptake but reduced doxorubicin loading and subsequent delivery, particularly when using plasma from healthy volunteers. **Conclusions**: These findings underscore the importance of EV source and protein corona composition in optimising drug delivery strategies. Our results suggest that autologous, patient-derived EVs hold potential as a targeted drug delivery system for CRC, but highlight the need for further optimisation of EV isolation, loading methods, and understanding of how disease progression affects EV functionality. This approach could ultimately reduce systemic toxicity and improve therapeutic outcomes for CRC patients.

## 1. Introduction

Colorectal cancer (CRC) remains the third most common cancer and second most common cause of cancer-related deaths worldwide [1,2], despite considerable efforts to develop better treatment approaches and to improve screening to enable earlier treatment. In early stages of disease, CRC is highly treatable with good outcomes; over 90% of patients have survival beyond 5 years. However, almost 50% of patients are diagnosed in later, more invasive stages of disease where outcomes are much poorer. At stage IV, 5-year survival rates are only around 8% [3], pointing to the urgent clinical need for better treatment options.

Current management of CRC frequently involves surgical resection, often alongside chemotherapy which can be both adjuvant and neo-adjuvant [4]. Current chemotherapy approaches lean into regimes incorporating 5-fluoruracil, oxaliplatin or irinotecan (e.g., FOLFOX and FOLFIRI), with the primary aim of blocking cancer cell proliferation within the tumour, but despite widespread use, these regimes come with significant limitations [4,5]. Because of their non-selectivity, rapidly dividing non-cancer cells are targeted as well as cancer cells, leading to a range of debilitating side effects which include nausea, fatigue, hair loss, and increased susceptibility to infections [5]. In many cases, this presents potentially life-threatening consequences, leading to a compromised quality of life and limiting the dose and duration of administration. As well as more effective treatments, therefore, there is an urgent need for more targeted, less toxic therapeutic strategies.

Extracellular vesicles (EVs) are nano-scale, membrane-enclosed particles released by all cells and found in all biofluids [6]. EVs contain a range of bioactive cargo, which includes proteins, lipids and RNA which can then be uptaken by other cells, leading to functional cargo delivery [7,8]. As a natural biological cargo delivery system, they are of growing interest as potential vehicles for the delivery of anti-cancer agents [9,10]. Moreover, they show organotropism [11,12] and cancer tropism [13], suggesting the selectively of cargo transfer that could be adopted for selective organ-specific, cancer-targeted delivery. Bioengineering approaches have therefore been developed in order to exploit this potential [14], building on the natural capabilities of EVs to stabilise, target and deliver anti-cancer agents. Tailoring the loading method to be either active and passive has been shown to enable loading of both hydrophobic and hydrophilic drugs into EVs [15,16], enabling chemotherapies currently employed in cancer treatment to be delivered with good efficiency into cancer cells, spheroid models, and pre-clinical animal model systems [17,18,19,20].

The source of EVs used for human therapeutic delivery is a key consideration when developing EV-based therapeutics for human administration. A number of clinical trials have shown safety, efficacy, and non-immunogenicity of EVs in a range of disease indications [21], with efforts well underway to develop large-scale production. However, EVs are highly heterogenous and populations of EVs are known to be highly immunogenic, raising potential concerns of allograft-like reactions if EVs from non-autologous sources are used, particular for the repeated administration likely required for cancer therapy [22,23]. This highlights the need for careful development of large-scale EV production for therapeutics. The demonstration of cancer tropism suggests that cancer-derived EVs may be the most effective route for anti-cancer therapies—and autologous patient-derived EVs have now been shown to augment immunotherapy treatment, including for CRC, in early clinical trials [24,25], suggesting that autologous EV administration has potential for therapeutic use; however few studies have explored the variables affecting loading and delivery of autologous patient-derived therapeutic EVs.

In this study, we assessed how disease stage alters loading and delivery characteristics using tracking of the fluorescent chemotherapeutic doxorubicin into EVs derived from plasma and tissue. Further, we assessed whether the protein corona known to coat the surface of circulating EVs impacted both loading and delivery [26], with the aim of assessing how these factors impact the utility of patient-derived, autologous EVs as a delivery system in cancer treatment.

## 2. Materials and Methods

### 2.1. Clinical Sample Collection and Processing

Clinical samples were collected from CRC patients at the Department of General Surgery, Sheffield Teaching Hospitals NHS FT, following written Research Ethics Committee approval (REC 19/NI/0221) and after obtaining full informed consent. All CRC patients admitted for colonic resection were considered eligible for the study. Blood was collected in EDTA vacutainers, and samples for the study were selected as either early stage (T2, no evidence of nodal (N) involvement or distant metastasis (M)) or late stage (T3-4, N1+ or M1). Healthy control blood was obtained under the same approvals and consent from volunteers at Sheffield Hallam University; any volunteers with a previous cancer diagnosis or a current pregnancy or illness were excluded from participation. Blood was processed to cell-free plasma within 3 h of collection by centrifugation in a Fisher FT 1R centrifuge at 1000 *g* for 10 min at 4 °C, with dilution of 1:1 in PBS, and further centrifugation of the supernatant first at 1500 *g* for 15 min at 4 °C, then at 2500 *g* for 15 min at 4 °C as previously described [27]. Plasma was then stored at −80 °C until use. Tissue samples were obtained from non-diseased sections of the colon following surgical resection of CRC patients. Samples were flash frozen at −80 °C until use, then defrosted into a digestion solution consisting of 5-fold *v/w* 1.5 mg/mL collagenase type I (Sigma-Aldrich, Poole, UK) in PBS, and underwent incubation at 37 °C for 1 h with constant agitation. Undigested material was then removed by centrifugation at 13,000 rpm for 30 min at 4 °C and supernatant then treated with protease inhibitors (Roche cOmplete, Sigma-Aldrich), before proceeding to EV isolation. Details of all patient samples used are provided in Table 1.

### 2.2. Cell Culture and Maintenance

The primary adenocarcinoma SW480 cell line and secondary adenocarcinoma SW620 cell line [28] were purchased from the European Collection of Authenticated Cell Cultures (ECACC), and cultured in complete Dulbecco’s Modified Eagle Medium (cDMEM), which contained high-glucose DMEM, GlutaMAX, pyruvate (Gibco, Thermo-Fisher, Hemel Hempstead, UK), 10% *v*/*v* foetal bovine serum (FBS, Gibco, Thermo-Fisher, UK) and 1% *v*/*v* penicillin/streptomycin (PS, Lonza Ltd., Slough, UK). Cells were maintained in a humidified incubator at 37 °C and 5% CO_2_ in air; culture medium was replaced every 3–4 days and cells sub-cultured at 70–80% of confluence and used between passage numbers 50–65. Cells were checked for mycoplasma with MycoAlert© Detection kit (Lonza Ltd., Slough, UK) every 6 months and were shown to be negative throughout.

### 2.3. CRC EV Isolation

SW480 and SW620 EVs were isolated as previously described [27]. Briefly, 2.5 × 10^7^ SW480 or SW620 cells were cultured in WHEATON^®^ CELLine™ AD-1000 Bioreactor flasks (DWK Life Sciences, GmbH, Wertheim, Germany) in 10% *v*/*v* Gibco EV-depleted FBS (Thermo-Fisher, UK) and DMEM, to avoid serum EV contamination. EV-enriched conditioning media (CM) were harvested weekly, alongside media replacement. Conditioning media were centrifuged at 300× *g* for 5 min and the supernatant centrifuged again at 2000× *g* for 5 min, then it was concentrated to reach a volume of 0.5 mL and ultrafiltered through Vivaspin^®^ 20 (100 kDa MWCO) (Sartorius, Göttingen, Germany) at 3000 *g* and 4 °C. For plasma and tissue EV isolation, 0.5 mL of processed sample was used for EV isolation. EVs from all sample types were separated from soluble factors by size-exclusion chromatography (SEC) loaded in Econo-Pac columns (Biorad, Watford, UK) with 10 mL sepharose CL-2B (GE Healthcare, Uppsala, Sweden) and eluted in PBS as 20 500 μL fractions. Protocols for removal of lipoprotein co-isolates were performed according to the published methodology; briefly, samples were brought to pH 5.5 using concentrated HCl [29] prior to SEC, or treated with styrene–maleic acid (SMA) at a 1:5 particle:SMA ratio [30].

### 2.4. EV Quantification and Nano-Flow Cytometry

Bicinchoninic acid (BCA) assay and Qubit HS RNA assay were performed according to manufacturer’s instructions (Thermo Fisher, Waltham, MA, USA). Relative levels of tetraspanin expression were assessed using a dissociation-enhanced lanthanide fluorescence immunoassay (DELFIA©, Revvity, Llantrisant, UK), as previously described [27], using antibodies to the canonical EV surface markers CD9 (Abcam, Cambridge, UK; 1:5000 dilution), CD63 (Bio-Rad Ltd., Watford, UK; 1:5000 dilution) and CD81 (Bio-Rad Ltd., Watford, UK; 1:5000 dilution), and to lipoprotein contaminants ApoA1 and ApoB (Proteintech Europe, Manchester, UK; 1:2000 dilution). Western blotting for CD9, CD81, TSG101 (Santa Cruz Biotechnology, Santa Cruz, CA, USA) and GM130 (Abcam, Cambridge, UK) using antibodies at 1:1000 dilution was undertaken on 30 μL EV samples after separation on 4–20% gradient gels as previously described [27], with transfer performed using Bio-Rad Turbo-blot and visualisation using Li-Cor HRP-conjugated secondary antibodies and SuperSignal Femto West ECL on a Li-Cor Odyssey XP instrument. EV size and particle count were assessed using a nanoanalyser instrument based on nano-flow cytometry (NanoFCM, MediCity, Nottingham, UK). EVs were compared to QC beads (250 nm silica standard) and size beads (68, 91, 113 and 155). Doxorubicin loading was assessed using the Phycoerythrin (PE) channel with a 580/40 nm bandpass filter.

### 2.5. Transmission Electron Microscopy

Transmission electron microscopy (TEM) of EVs was performed at the Electron Microscopy facility, Faculty of Science, University of Sheffield. EVs were transferred through absorption onto carbon-coated copper grids for 1 min, quickly dried with filter paper and then rinsed twice with water. EVs were stained for 2 min in filtered 2% uranyl acetate. The grid was allowed to dry for 10 min before staining in uranyl formate. Grids were visualised on an FEI Tecani G2 Spirit BioTwin (PennState, University Park, PA, USA) TEM, and images were recorded using a Gatan Orius 1000B CCD camera and Gatan Digital Micrograph software (v 3.5, Gatan, Pleasanton, CA, USA).

### 2.6. Doxorubicin Loading and Flow Cytometry Analysis

Bioreactor- and plasma-derived EVs at 100 µg/mL protein concentration obtained by BCA assay were co-incubated with doxorubicin (Sigma-Aldrich, Poole, UK) at 100 µg/mL for 2 h at 37 °C, as previously described [17], on a shaker (20 oscillations per minute). Where necessary, EVs were then also labelled with Celltracker CMFDA (Thermo Fisher Scientific, Waltham, MA, USA) at a final concentration of 20 µM for 45 min at 37 °C in order to co-analyse EV delivery and drug delivery. Samples were ultracentrifuged at 100,000 *g* for 1 h to pellet EVs, with the supernatant removed and the EVs resuspended in PBS prior to a second ultracentrifugation step to wash the EVs. Efficiency of drug loading was determined by measuring doxorubicin fluorescence (FL Exc 590 nm/Em 615 nm) in the pelleted EVs (EV) and the supernatant (SN) using a BMG Clariostar instrument, and calculated as [FL EV/(FL EV + FL SN) × 100]. EVs were then suspended in 200 μL PBS in preparation for cell treatments. SW480 and SW620 cells were seeded at 2.5 × 10^5^ cells/mL in 24-well plates (Nunc) and incubated for 24 h at 37 °C (5% CO_2_). Media were removed and replaced with media containing 30 µg/mL doxorubicin-loaded EVs, and cells were incubated for a further 18 h. For analysis, media were removed, and cells were washed with PBS and trypsinised. Cells were centrifuged at 1000× *g* for 5 min, the supernatant was removed, and cells were resuspended in PBS. Cells were analysed by flow cytometry (Cytoflex, Beckman-Coulter, Brea, CA, USA). Cells were first gated using forward scatter and side scatter to identify cells and eliminate cell debris, then loading-quantified using the PE channel (doxorubicin) and FITC channel (Celltracker CMFDA) with unloaded, untreated cells as label-free controls.

### 2.7. Protein Corona Manipulation and Assessment by SDS-PAGE

Protein corona involvement was assessed using two methods. Firstly, 100 μg/mL of SW480/SW620-derived EVs was treated with 1 mL EV-depleted plasma protein (fractions 12–20) obtained following the SEC separation for 48 h at 4 °C, prior to pelleting by ultracentrifugation at 100,000 *g* for 1 h at 4 °C. Secondly, the protein corona of plasma-derived EVs was degraded by incubation with 25 μg/mL proteinase k and 100 μg/mL trypsin for 1 h at 37 °C, before stopping the reaction using protease inhibitors (cOmplete, Roche, Basel, Switzerland) and pelleting by ultracentrifugation. The impact of treatments on EV protein content was assessed by SDS-PAGE. Briefly, 20 μL of treated or untreated EVs was loaded onto 10% polyacrylamide resolving gels, and electrophoresis was performed at 180 V for 1 h. Gels were then stained with 0.25% (*w*/*v*) Coomassie Brilliant Blue R-250 (Sigma-Aldrich, St. Louis, MO, USA) in 40% methanol and 10% acetic acid. Images of the gels were than captured after brief destaining in methanol and acetic acid.

### 2.8. Viability Assay After EV/Drug Treatments

The response to EV/drug loading was performed using Alamar Blue assays as previously described [17]. SW480 and SW620 cells were seeded at 5 × 10^3^ cells/mL in a Nunc 96-well plate and incubated at 37 °C for 24 h, before treatment with or without EVs (30 μg/mL), and compared to a dose–response curve of doxorubicin alone. Cells were incubated for 48 h at 37 °C, then analysed by addition of 30 μg/mL resazurin sodium salt for 4 h before reading at Ex/Em 530-560/590 on a Clariostar plate reader (BMG Labtech, Aylesbury, UK). Lactate Dehydrogenase (LDH) assays were performed using the CyQuant assay kit (Thermo Fisher), according to manufacturer’s instructions.

### 2.9. Statistical Analysis

All statistical analyses were performed using Prism 8.1.1. Flow cytometry data were analysed using FlowJo software (Version 7.0, FlowJo, Ashland, OR, USA), and % positive/mean Relative Fluorescence Unit (RFU) values were extracted from a minimum of 10,000 events. Data were tested for normality (gaussian distribution) with two modalities: D’Agostino & Pearson test and Shapiro–Wilk test. Multiple comparison through ordinary one-way analysis of variance (ANOVA) with Tukey’s test for post hoc analysis was performed for parametric data, while the Kruskal–Wallis test with Dunn’s test for post hoc analysis was performed for non-parametric data. For comparison of two groups, a *t*-test (parametric) or Mann–Whitney test (non-parametric) was used. *p*-values < 0.05 were considered significant, and data are illustrated as mean ± standard error of the mean (SEM).

## 3. Results

### 3.1. EVs with Distinct Profiles Isolated from Cell and Patient Sources

**Figure 1:**
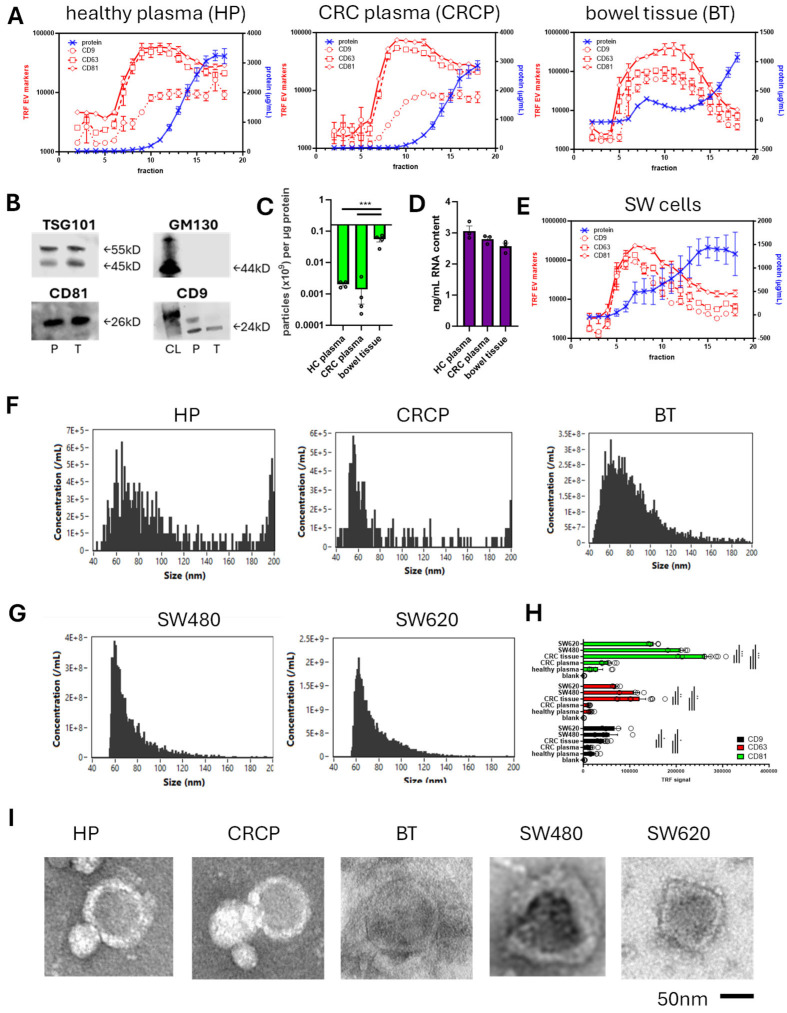
Isolation and characterisation of CRC-derived EVs. For the isolation of patient-derived EVs, isolations were undertaken from plasma (healthy plasma, HP; CRC plasma, CRCP) and bowel tissue (BT) by SEC. Isolation profiles from SEC were monitored using BCA assay and DELFIA ELISA detection of canonical EV markers ((**A**), n = 4). EVs pooled from fractions 6–11 were then assessed by Western blotting for positive (luminal TSG101, and membrane CD81 and CD9) markers and the negative golgi marker GM130 ((**B**), showing P = plasma isolation, T = tissue isolation, CL = 2 μg SW620 cell lysate control). EV pools from these clinical samples were assessed by particle/protein ratio ((**C**), n = 4), and by RNA content ((**D**), n = 4). For comparison, EVs were also isolated from SW480 and SW620 cells ((**E**), showing SW480 elution profile) as previously described and characterised [27]. Analysis of size profiles was assessed using nano-flow cytometry of clinical sample-derived EVs (**F**) and SW cell-derived EVs (**G**), and expression of the canonical markers in the EV pools was confirmed by DELFIA ELISA comparison across all the EV sources ((**H**), n = 4–8 per group). Finally, presence of membrane-enclosed EVs was confirmed using TEM (**I**). * = *p* < 0.05; ** = *p* < 0.01; *** = *p* < 0.0001.

**Figure A1:**
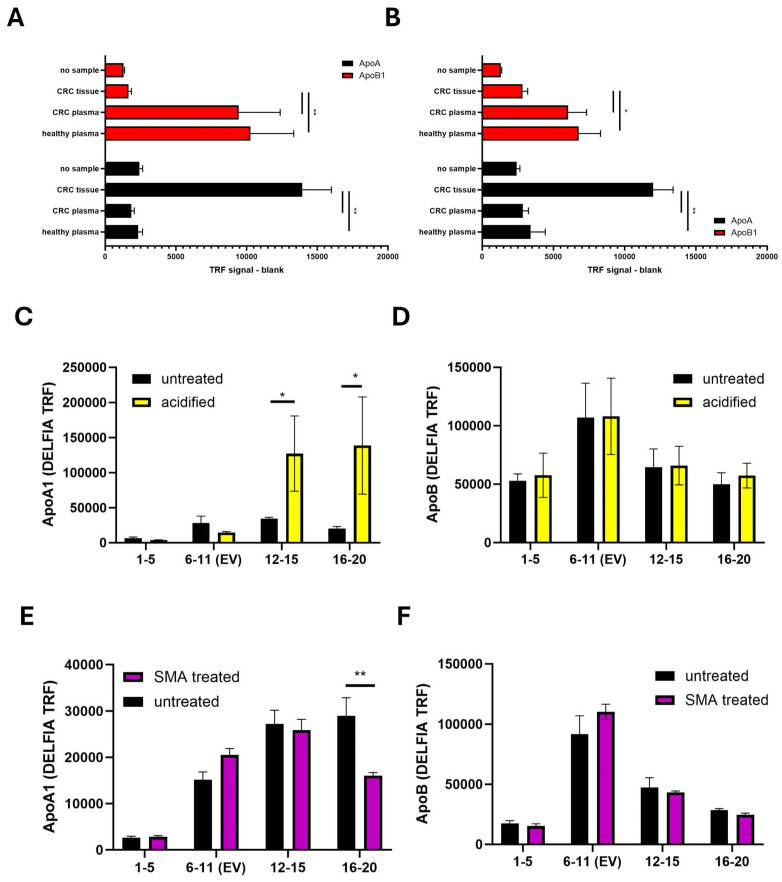
Presence of apolipoproteins in EV-enriched SEC fractions 6–11 (**A**) and protein-rich fractions 12–15 (**B**), with higher levels of ApoA1 co-isolation in plasma EVs, and higher levels of ApoB co-isolation with tissue EVs. Acidification of EVs prior to isolation using HCl to obtain pH5.5 did not significantly alter the lipoprotein content in EV-enriched fractions 6–11, although more ApoA1 was observed in the protein-rich later fractions (**C**,**D**). Treatment of EVs with styrene–maleic acid (SMA) also did not alter lipoprotein content in EV-enriched fractions 6–11 (**E**,**F**). * = *p* < 0.05, ** = *p* < 0.01 (n = 4 per group).

In order to generate EVs for use in drug loading studies, we assessed EV isolation from the different EV samples used in the study, enabling us to characterise and compare our isolation methods across EVs from different CRC sources. Using SEC, EVs from samples were eluted in 20 × 0.5 mL fractions, enabling the tracking of the elution profile. EVs from plasma and tissue samples showed protein EVs beginning to elute at fraction 5–6, as assessed using ELISA for the canonical markers CD9, CD63 and CD81 (Figure 1A). These markers all co-isolated, indicating an EV population containing all of these markers. In contrast, protein, as assessed by the BCA assay, eluted more prominently from all samples in fractions 12–20, suggesting successful separation of EVs from free protein. Tissue samples did show a small peak of proteins in the EV-rich fractions 6–11, which we have previously observed from cell culture EVs using this method [17,27], but protein was undetectable in the EV-rich fractions of plasma. No obvious differences in elution profile were observed between plasma from healthy volunteers and CRC patients (Figure 1A). As it is a common issue with isolation from clinical samples, we anticipated significant non-EV co-isolates of lipoproteins, and using DELFIA ELISA observed some co-isolation of apolipoprotein (Apo)B in tissue and some co-isolation of ApoA1 in plasma samples (Appendix A, Figure A1A,B). To address this, we attempted published protocols for removing lipoproteins, including acidification (Appendix A, Figure A1C,D [29]), and treatment with styrene–maleic acid (SMA; Appendix A, Figure A1E, F [30]). In both cases, although a small, non-significant decrease in ApoA1 was observed in acidified EVs (Appendix A, Figure A1C), we did not achieve significant reduction and therefore did not continue with these methods.

**Figure A2:**
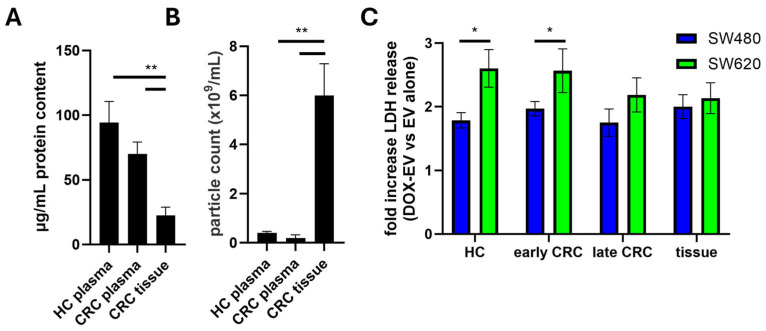
Characterisation and treatment of EVs. EVs isolated from plasma had higher protein content compared to EVs isolated from tissue (**A**), but there was no difference between healthy and CRC plasma (n = 4–6 per group). Significantly higher particle yields were obtained from tissue compared to plasma (**B**), but there were no differences between healthy and CRC plasma. Treatment of SW480 and SW620 cells with DOX-loaded EVs resulted in statistically significantly higher LDH release from SW620 cells compared to SW480 cells for healthy and early CRC plasma (**C**). * = *p* < 0.01, ** = *p* < 0.01.

The elution profile of EV markers compared to free protein led us to pool fraction 6–11 for drug loading purposes, and assessing pooled samples by Western blotting showed that this SEC isolation method obtained pools enriched for the luminal marker TSG101 and the surface markers CD81 and CD9, and absent from the cellular golgi marker GM130 (Figure 1B). We further assessed the pooled EVs by comparing protein concentration (Appendix A, Figure A2A) and particle counts (Appendix A, Figure A2B) between groups, and lower protein content coupled to higher particle counts showed significantly higher purity of tissue-derived EVs compared to plasma as assessed using the particle/protein ratio (Figure 1C, *p* < 0.0001). No differences were observed measuring RNA content between plasma and tissue samples (Figure 1D). For comparison with clinical samples, we also isolated EVs from SW480 and SW620 cell lines using bioreactor culture, as previously fully characterised in our group [17,27]. The elution profile was consistent with our previous work and similar to the clinical samples (Figure 1E). Analysis of size distribution and particle counts using nano-flow cytometry showed particles ranging predominantly between 40 and 120 nm (Figure 1F), with lower yields observed in plasma (in the range of 1 × 10^6^/mL, as compared to 1 × 10^8^/mL for tissue and 1 × 10^9^/mL for SW cells; Figure 1G) which suggests that yield is why the protein concentration is low in plasma EV fractions (Figure 1A). All EV pools expressed canonical EV markers CD9, CD63 and CD81 with differences observed using DELFIA ELISA, presumably supporting differences in EV yield (Figure 1H). Finally, EV pools were analysed by TEM, showing double-membrane enclosed structures in an appropriate size range consistent with our previous observations [17,27] in the case of plasma frequently co-localising with what may be lipoproteins (Figure 1I).

### 3.2. Patient-Derived EVs Have Distinct Doxorubicin Loading Kinetics

**Figure 2:**
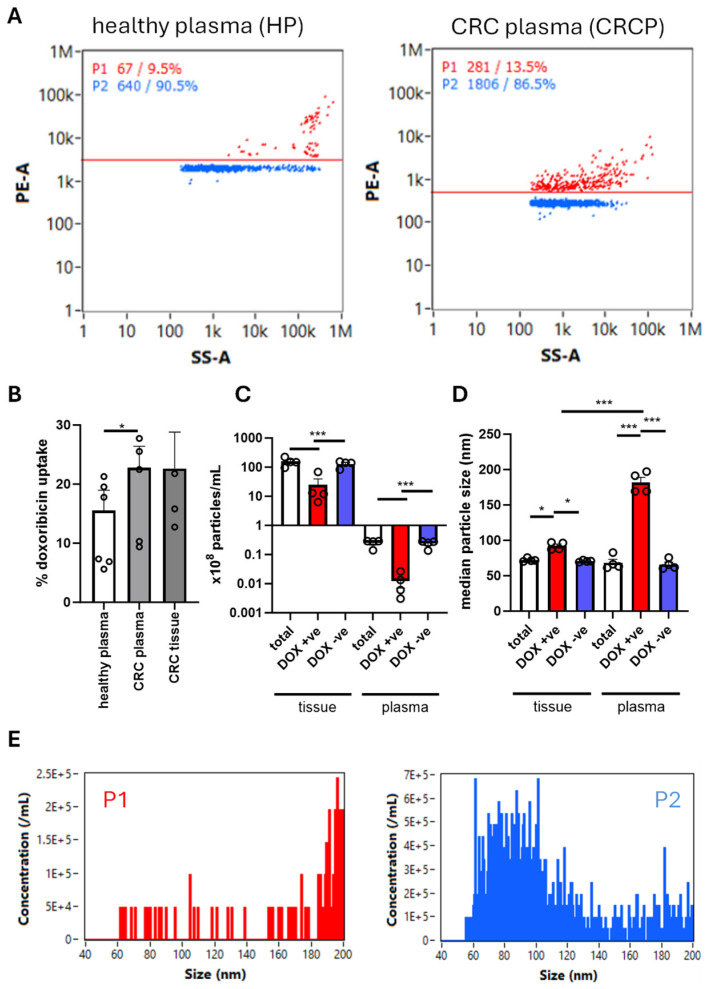
Loading of CRC-derived EVs with doxorubicin. EVs were loaded with doxorubicin using passive incubation, which was monitored by tracking EVs on the PE channel using nano-flow cytometry identifying the positive (P1) and negative (P2) populations (**A**). Efficiency of doxorubicin loading was calculated following fluorescence measurement of the EV-loaded drug compared to the total drug ((**B**), n = 4–6 per group), then nano-flow cytometry enabled particle counts to be assessed in the P1 and P2 populations ((**C**), n = 4 per group), as well as particle sizes ((**D**), n = 4 per group), with representative size profile plots of P1 and P2 also shown (**E**). * = *p* < 0.05; *** = *p* < 0.0001.

After showing successful isolation of EVs from clinical samples, we next assessed the efficiency of loading doxorubicin. Overall loading efficiency was not high, in agreement with our previous work [17], ranging between 8 and 30% (Figure 2). Interestingly, drug loading into plasma-derived EVs from CRC patients was significantly higher than loading into plasma from healthy volunteers (Figure 2A–C, *p* < 0.05). We also observed that loading into CRC plasma-derived EVs was similar to that of loading into tissue-derived EVs (Figure 2B), at around 22%, although the higher yield of EVs from tissue did lead to higher overall yields of doxorubicin-positive EVs (Figure 2C, *p* < 0.0001). Finally, doxorubicin-loaded particles from both plasma and tissue were larger compared to the total EV population (Figure 2D, *p* < 0.05), considerably so when considering plasma samples where doxorubicin-loaded particles had a median size of almost 200 nm, compared to 60–70 nm for unloaded particles (Figure 2D, *p* < 0.0001, with representative plots shown in Figure 2E). Overall, we observed successful drug loading into patient-derived EVs sourced from both plasma and tissue, but with distinct dynamics towards higher drug loading in EVs from CRC patients, higher yields of loaded EVs from tissue and a tendency towards loading of larger EVs.

### 3.3. CRC Stage Influences the Cellular Delivery of Patient-Derived EVs

**Figure 3:**
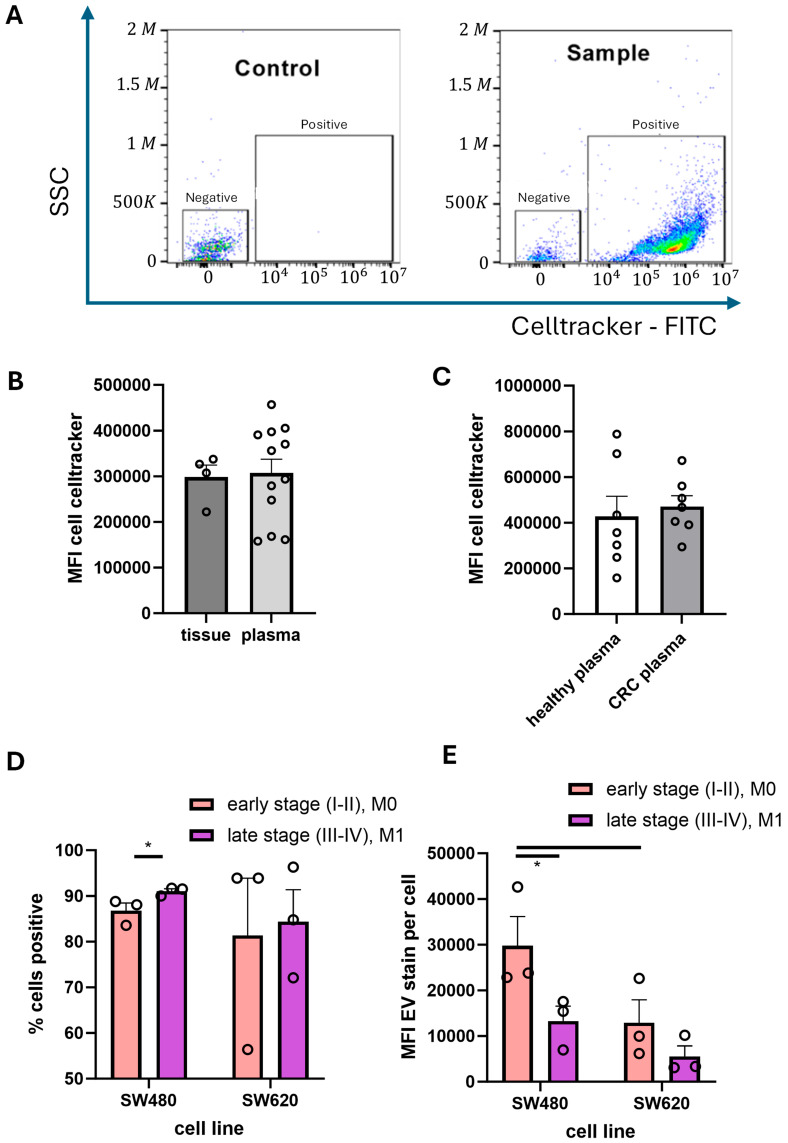
Loading of CRC-derived EVs to CRC cells. EV delivery was monitored following labelling with Celltracker CMFDA using the FITC channel of a CytoFlex flow cytometer ((**A**), showing representative images from SW480 cells). Mean fluorescence intensity was collected after separating the positive and negative cell populations ((**B**), n = 4–12 per group and (**C**), n = 6–7 per group), and the percentage of positive cells calculated after treatment with EVs from different stages of disease delivered into SW480 and SW620 cells ((**D**,**E**), n = 3 per group). * = *p* < 0.05.

After showing successful loading of EVs, we next examined whether patient-derived EVs could be delivered into recipient cancer cells. We used the isogenic SW480 and SW620 cell line model; since SW480 was isolated from a primary tumour and SW620 from a patient-matched lymph node metastasis, they provide a validated model of CRC progression [28]. EVs were labelled with a Celltracker dye enabling their detection using FITC settings, with a clear shift observed after cell treatment with labelled EVs (Figure 3A). No significant differences in EV delivery were observed when comparing uptake of plasma-derived EVs to tissue-derived EVs (Figure 3B), or when comparing uptake of plasma-derived EVs from healthy volunteers to CRC patients (Figure 3C). Very high delivery of patient-derived EVs was observed, with 80%+ positivity (Figure 3A,D). We next assessed whether disease stage influenced EV delivery. A small but significant increase in the percentage of delivery was observed for EVs derived from later stage disease (Figure 3D, *p* < 0.05). Interestingly, when using mean fluorescence intensity (MFI) which allowed us to separately assess whether more EVs were uptaken as opposed to percentage positive which only assessed whether more cells had taken up any EVs, significant differences related to disease stage were observed. More EVs were uptaken by the primary cell line SW480 compared to the metastatic cell line SW620 (Figure 3E, *p* < 0.05). We also observed that plasma-derived EVs from late-stage CRC were uptaken less than EVs from early-stage CRC—this was statistically significant for delivery into SW480 cells (Figure 3E, *p* < 0.05). Overall, the data relating to patient-derived EV uptake into cancer cells were dependent on disease stage in terms of both donor EV and recipient cell.

### 3.4. CRC Stage Influences Cellular Delivery of Doxorubicin from EVs and Subsequent Viability

**Figure 4:**
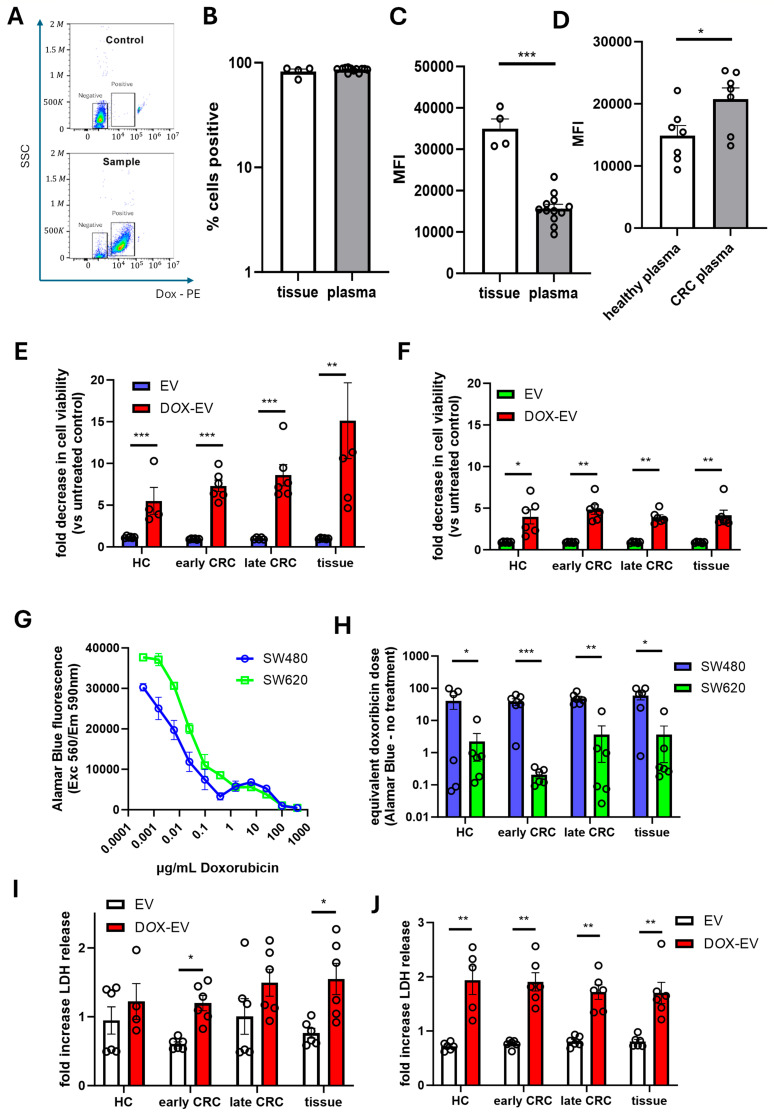
Delivery of doxorubicin from drug-loaded CRC-derived EVs. Delivery of doxorubicin was assessed by monitoring the PE channel on the CytoFlex flow cytometer ((**A**), showing representative plots of SW480 cells). This allowed calculation of % doxorubicin-positive cells ((**B**), n = 4 per group) and mean fluorescence intensity ((**C**,**D**), n = 4–12 per group) following treatment of cells with patient-derived EVs from different sources. The impact of this drug delivery on cell viability was then assessed using Alamar Blue viability assay on treated SW480 ((**E**), n = 4) and SW620 ((**F**), n = 4) cells, with data plotted as the fold change in fluorescence compared to untreated (no EV treatment) control wells for unloaded (EV) and loaded (DOX-EV) treatments. These data were then calibrated to a dose–response curve generated using serial dilutions of doxorubicin (**G**) to calculate an effective dox-equivalent dose (**H**). LDH release from treated SW480 ((**I**), n = 4) and SW620 ((**J**), n = 4) cells was also measured. * = *p* < 0.05; ** = *p* < 0.01; *** = *p* < 0.0001.

Following our investigations of EV uptake, we next assessed how EV uptake translated into delivery of a chemotherapeutic, thus connecting efficiency of drug loading to EV uptake. Adapting the flow cytometry method to use the PE channel, doxorubicin loading into cells was assessed, with a high level of doxorubicin-positive cells observed after DOX-EV treatment (Figure 4A) correlating with the high EV-positive cell percentage previously seen (Figure 3A). No differences were observed in the number of DOX-positive cells when comparing tissue to plasma-derived EVs (Figure 4B); however when using MFI to assess the amount of EVs taken up by individual cells, tissue EVs showed significantly higher delivery (Figure 4C, *p* < 0.0001). Comparing doxorubicin delivery between plasma-derived EVs from healthy volunteers to CRC patients, CRC-derived EVs showed significantly higher levels of delivery (Figure 4D, *p* < 0.05). Therefore, we observed good chemotherapy delivery which was higher in CRC-derived EVs, which presumably was due to better doxorubicin loading (Figure 2B) rather than better EV delivery (Figure 3A–C).

Finally, we assessed how this EV delivery translated into cell viability in response to the EV-delivered therapeutic agent. Using an Alamar Blue assay, we observed that DOX-EVs derived from patient samples all significantly decreased the viability of both SW480 (Figure 4E) and SW620 cells (Figure 4F), compared with EVs with no drug loading (*p* < 0.0001). An incremental increase in response was observed with SW480 cells, from healthy plasma to early CRC, late CRC and then tissue EV (Figure 4E), although this was not significant, however SW620 cells remained relatively consistent regardless of EV source (Figure 4F). Using a dose–response curve generated from free doxorubicin (Figure 4G), we were able to show that SW480 cells were significantly more vulnerable to doxorubicin-derived EVs than the metastatic SW620 cells (Figure 4H, *p* < 0.0001), with delivery of DOX-EVs achieving effective responses equivalent to delivery of 38–59 μg/mL of doxorubicin for SW480 cells depending on EV source, compared with only 0.2–3.6 μg/mL for SW620 cells. Finally, we complemented Alamar Blue measurements by measuring LDH release from cells after EV treatment, observing a pattern similar to the Alamar Blue data, with DOX-EVs increasing LDH release compared to unloaded EVs (Figure 4I,J); interestingly, higher LDH release was observed from SW620 cells compared to SW480 cells (Appendix A, Figure A2C).

### 3.5. Protein Corona Alters Delivery of DOX-EV

**Figure 5:**
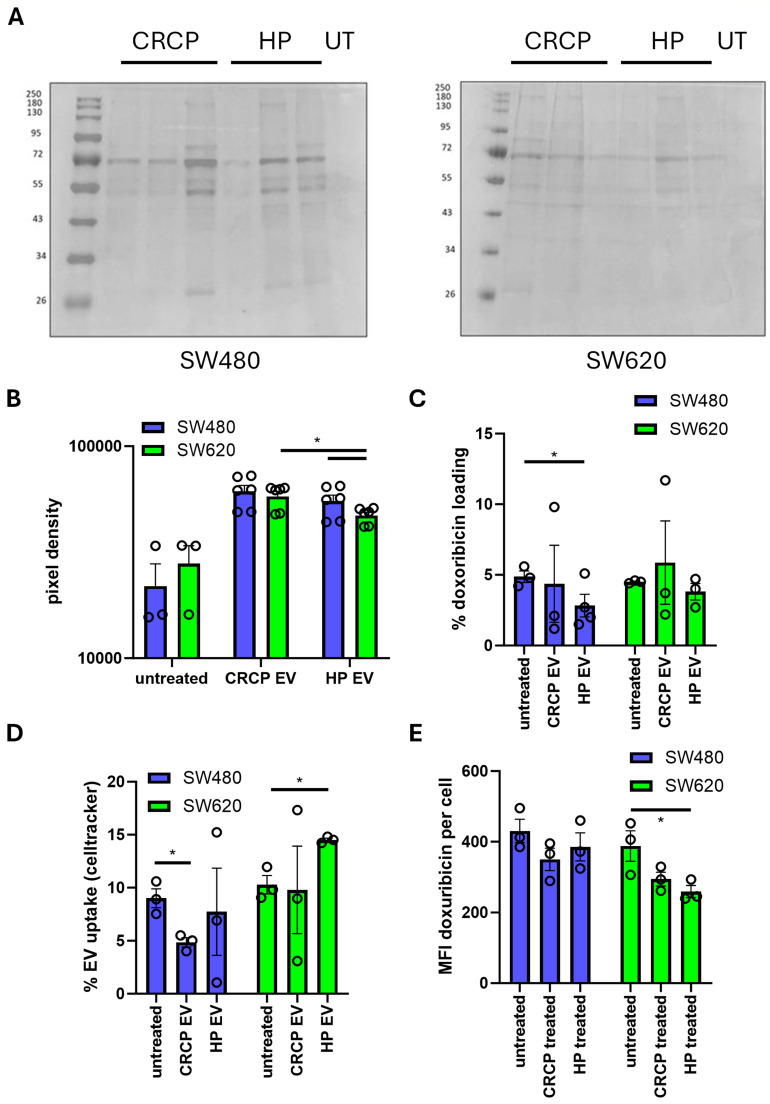
Impact of plasma protein treatment of EVs on EV/doxorubicin delivery. EVs derived from cell lines SW480 and SW620 were treated with plasma proteins (**A**). Association of proteins with EVs was assessed using SDS-PAGE (**A**) and quantified using densitometry ((**B**), n = 3–6 per group). Loading of doxorubicin was then assessed on these EVs using nano-flow cytometry ((**C**), n = 3–4), and after EV treatment of cells, the percentage of EV delivery assessed by monitoring Celltracker CMFDA delivery (**D**) and doxorubicin delivery (**E**) using flow cytometry. * = *p* < 0.05.

**Figure A3:**
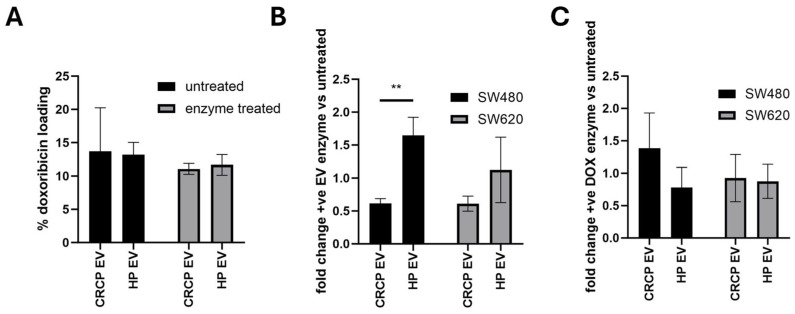
Treatment of EVs with proteases did not alter doxorubicin loading ((**A**), n = 3). Significantly higher delivery of DOX-EVs was observed when treating SW480 cells, with healthy plasma-derived EVs compared to CRC EVs ((**B**), n = 3). No differences were observed in DOX-EV delivery after protease treatment ((**C**), n = 3). ** = *p* < 0.01.

Finally, it is becoming increasingly clear that EVs derived from patients carry a protein corona on their surface which is involved particularly in EV uptake [26]. We observed a high percentage of EV delivery from patient-derived EVs compared to our previous work using cell line-derived EVs [17]. We therefore performed experiments to explore whether the protein corona could influence dox-EV delivery. This was done firstly by exposing cell line-derived EVs to plasma-derived proteins to allow corona formation, and conversely by trying to strip surface proteins using enzymes. Enzymatic treatment did not significantly impact DOX-EV delivery (Appendix A, Figure A3A), although EV delivery was affected differently when comparing healthy plasma-derived EVs and CRC plasma-derived EVs (Appendix A, Figure A3B). Treatment of SW cell-derived EVs did increase the presence of EV-associated protein particularly when plasma protein was used (Figure 5A); association of proteins with SW480 EVs was higher than with SW620 EVs (Figure 5B, *p* < 0.05) and there was significant inter-patient variability in how well EVs associated with plasma protein. These protein-exposed EVs were then loaded with doxorubicin. Interestingly, much lower loading efficiency was observed for these cell-derived EVs compared to patient-derived EVs (Figure 3)—more consistent with our previous observations [17]—and the treatment with plasma protein from healthy volunteers, which showed the highest association of protein with EVs, significantly decreased the efficiency of loading (Figure 5C, *p* < 0.05). Next, these EVs were used to treat SW480 and SW620 cells. Loading with EVs treated with plasma protein from healthy volunteers showed higher delivery of EVs to both cells compared to EVs treated with CRC plasma protein. When treating the SW620 cells, EVs treated with healthy plasma protein were uptaken significantly more compared to untreated EVs (Figure 5D, *p* < 0.05). Finally, doxorubicin delivery was assessed, and treatment with plasma protein did not significantly alter delivery to SW480 cells, but treatment with healthy plasma protein significantly reduced doxorubicin delivery compared to controls. Overall, plasma protein pre-treatment impacted doxorubicin loading of SW480 EVs more readily associated with plasma proteins particularly from healthy volunteers, and whilst this protein pre-treatment enhanced EV delivery, it reduced doxorubicin delivery, which presumably is due to the impact on the ability to load doxorubicin rather than deliver EVs themselves.

## 4. Discussion

In this study, we assessed whether autologous EVs could be a viable strategy for delivery of cancer treatment, building on growing interest in using EVs as drug delivery vehicles [9,10] and addressing the potential issues that may be caused by use of non-autologous EVs in terms of inducing an allograft rejection-type response with repeated administration [22,23]. Despite the enormous promise of EVs in clinical applications, progress is hampered by significant regulatory challenges related to the technical challenges in isolation, quality control, and storage, and progressing towards demonstration of safety and efficacy [31]. The use of autologous EVs in this space provides an approach similar in principle to the development of CAR-T therapy [32] and thus provides a possible alternative to large-scale cell-based manufacturing by moving towards clinical adoption of EV therapeutics in cancer treatment if issues around scalability and standardisation can be overcome. Given that cancer-derived EVs show cancer tropism [11,12,13], an autologous EV delivery vehicle could also increase specificity, decreasing systemic toxicity and increasing tolerance levels to chemotherapies which currently have significant debilitating side-effects [5]. We used doxorubicin for this study as it is fluorescent, making it easy to detect on both flow cytometry and nano-flow cytometry platforms; doxorubicin itself shows high system toxicity particularly to the cardiovascular system, and as such is rarely used for CRC [33]. Future work building on this autologous EV approach will use more clinically relevant cargo, such as oxaliplatin, which we have previously explored using cell line-derived EVs [17].

There is a growing body of work exploring methods for bioengineering drugs into EVs, with a number of loading methodologies now tested in comparative studies [15,16]. Recent work has suggested that active loading methods (e.g., using sonication or freeze–thawing) may enhance loading into plasma-derived EVs [16]. We observed loading efficiencies of around 20–30% for both plasma- and tissue-derived EVs, so exploring alternative loading methods to build on this work using passive incubation could deliver significant benefits to loading efficiency. Interestingly, CRC-derived EVs were loaded more efficiently than EVs from healthy volunteers—this demonstrates a need for careful consideration of EV source when developing autologous delivery. However, in this proof-of-concept, small-group study, our healthy controls were significantly younger than patients due to the local availability of donors. Age substantially alters EV cargo and effects [34], so this is a significant confounding factor which we are now trying to address by collecting plasma from older volunteers. Since first-line treatment of CRC tends to involve surgical resection, there is the potential in this disease to use tissue EVs as a resource—our data indicate that tissue EVs load as efficiently as plasma-derived EVs but their yield is much higher, which could be valuable when it comes to clinical use and the need for sufficient EVs to be effective.

Another observation made in this work is that CRC stage has a significant impact on the ability to deliver drug-loaded EVs. Particularly, delivery of doxorubicin into the metastatic SW620 cells was lower than delivery into the primary SW480 cells. Additionally, in terms of therapeutic benefit, by using a doxorubicin dose–response calibration curve, we were able to observe responses equivalent to 38–59 μg/mL doxorubicin for SW480 cells which suggests high-efficiency delivery given that we achieved 20–30% EV loading efficiency from 100 μg/mL doxorubicin stock, and used each preparation for eight technical replicates. By contrast, delivery to the metastatic SW620 achieved much lower efficacy, achieving responses comparable to 0.2–3.6 μg/mL, pointing to the challenge in treating CRC cells in an advanced diseased stage. In addition to this, EVs from later stages of disease (defined here as invasive characteristics—nodal involvement or presence of distant metastasis) showed poorer delivery. This points to a challenge in delivering drugs to the stages of disease most at clinical need of new approaches. A number of factors are known to influence EV uptake, including surface proteins [11,35] and tumour microenvironment properties including mechanical stiffness [36]. We did not determine the mechanism of EV uptake in this work, but multiple modalities have been described and methods exist to explore them using inhibitors of key mediators [37]. Studies are needed to unpick whether different mechanisms (e.g., membrane fusion vs. endocytosis) play a role depending on surface characteristics and/or EV source. Understanding and leveraging this may then enable us to enhance the natural delivery properties of EVs using bioengineering to overcome the limitations of targeting to advanced-stage disease where clinical need is greatest [14].

Since we observed much higher delivery efficiency of patient-derived EVs compared to cell line-derived EVs, we considered that the protein corona known to coat circulating EVs could be an important factor in delivery [26,36,38]. Since SEC is known to strip the “soft corona” from EVs (whilst leaving the “hard corona” intact [38]), we took advantage of fraction collection, using the protein-rich fractions from plasma to enable reformation of a protein coating in the EV-rich fractions from cell culture EVs. Protein staining seemed to show this was successful to an extent—although there was variability across individuals. Interestingly, our experiments using EV treatment with proteins derived from plasma suggested that protein coating reduces the efficiency of drug loading which directly translates into reduced delivery into CRC cells. This suggests another important variable in the use of patient-derived EVs. We did not assess the protein corona of tissue-derived EVs due to limited material available, but we are currently building a bank of samples that will enable us to look more closely at EV source in order to optimise drug loading. Given the growing understanding of the EV surface, ongoing work to understand coronal proteins and surface glycans will be crucial to improve EV therapeutic delivery. Given significant inter-patient variability observed in these experiments, future work with increased numbers of patients and detailed characterisation should provide a clearer picture.

Another issue when considering EVs derived from patients is the purity of the EV preparation; we have used SEC which is a common method for generating good quality and yield of EVs, but contamination with lipoproteins is a persistent problem due to their co-isolation in size- or density-based methods [39]. Indeed, our TEM images appear to show the presence of non-EV particles in our EV preparations, and we measured both ApoA1 and ApoB co-isolation in our clinical samples. Lipoproteins vastly outnumber EVs in plasma, and as such interfere substantially with isolating pure EVs, impact effective characterisation, and compromise standardisation [40]. This likely explains why plasma EVs appear to have poorer purity in this study using particle/protein ratio compared to tissue [41]. A number of methods have been proposed to remove lipoproteins from EV preparations [29,30,42,43]; acid treatment or treatment with SMA did not work in our hands but more work is needed on this in order to have confidence that the EVs are being loaded, rather than other particles which may not have the specificity or targeting that EV-based delivery systems could deliver. This is likely to be a central challenge to the adoption of EVs derived from autologous clinical material as therapeutics and the next generation of EV isolation methods are likely to play an important role [37].

## 5. Conclusions

Overall, we have shown that patient-derived EVs could form the basis of an autologous chemotherapy delivery vehicle due to the inherent and engineerable properties of EVs, building on recent studies developing autologous EVs to enhance other anti-cancer approaches [24,25]. In order to build on this proof-of-concept study, our future work will now be focused on full chemical characterisation of EV loading of different drugs and EV uptake mechanisms, improved EV isolation and removal of lipoprotein contaminants, increasing sample sizes and improving age matching to better characterise patient variability and the impact of disease stage, and understanding and leveraging surface profiles including protein and glycan corona. This will therefore begin moving this platform towards in vivo validation and pre-clinical testing for application in advanced stages of disease where there is an urgent unmet clinical need.

## Figures and Tables

**Table 1:** Demographic data for clinical samples used in the study.

Sample Type	n	Age Range (Mean)	Sex Ratio (F/M)
Early-stage CRC plasma (T2, N/M 0)	6	42–83 (68.3)	2/4
Late-stage CRC plasma (T3/4, N1+ or M1)	7	46–80 (66)	4/3
Healthy control plasma	9	24–59 (42.3)	7/2
Bowel tissue (T3)	4	43–79 (64.5)	2/2

## Data Availability

The original contributions presented in this study are included in the article. Further inquiries can be directed to the corresponding author.

## Abbreviations

The following abbreviations are used in this manuscript:

CRC	Colorectal Cancer
EV	Extracellular Vesicle
ECACC	European Cell and Culture Collection
DMEM	Dulbecco’s Modified Eagle Medium
FBS	Foetal Bovine Serum
PS	Penicillin and Streptomycin
CM	Conditioned Medium
SEC	Size-Exclusion Chromatography
SMA	Styrene–maleic Scid
BCA	Bicinchoninic Acid
TEM	Transmission Electron Microscopy
SN	Supernatant
LDH	Lactate Dehydrogenase
RFU	Relative Fluorescence Unit
ANOVA	Analysis of Variance
SEM	Standard Error of the Mean
HP	Healthy Plasma
CRCP	Colorectal Cancer Plasma
BT	Bowel Tissue
MFI	Mean Fluorescence Intensity
DOX	Doxorubicin
Apo	Apolipoprotein
